# Chronic obstructive pulmonary disease alters the genetic landscape and tumor immune microenvironment in lung cancer patients

**DOI:** 10.3389/fonc.2023.1169874

**Published:** 2023-06-14

**Authors:** Qiurui Zhang, Xijia Feng, Weiting Hu, Chengqiang Li, Debin Sun, Zhao Peng, Shengzhou Wang, Hecheng Li, Min Zhou

**Affiliations:** ^1^ Department of Pulmonary and Critical Care Medicine, Ruijin Hospital, Shanghai Jiao Tong University School of Medicine, Shanghai, China; ^2^ Institute of Respiratory Diseases, Shanghai Jiao Tong University School of Medicine, Shanghai, China; ^3^ Shanghai Key Laboratory of Emergency Prevention, Diagnosis and Treatment of Respiratory Infectious Diseases, Shanghai, China; ^4^ Department of Thoracic Surgery, Ruijin Hospital, Shanghai Jiao Tong University School of Medicine, Shanghai, China; ^5^ Genecast Biotechnology Co., Ltd., Wuxi, Jiangsu, China

**Keywords:** lung cancer, chronic obstructive pulmonary disease, genetic mutations, PI3K-Akt signaling pathway, tumor immune microenvironment

## Abstract

**Background:**

Chronic obstructive pulmonary disease (COPD) and lung cancer are leading causes of morbidity and mortality worldwide. Studies have reported molecular alterations in patients with lung cancer and in patients with COPD. However, few investigation has been conducted on the molecular characteristics of lung cancer patients with COPD.

**Materials and methods:**

We performed a retrospective cohort study that included 435 patients with pathologically confirmed lung cancer at the Ruijin Hospital. For patients with documented spirometry, Global Initiative for Chronic Obstructive Lung Disease criteria were used to define COPD. For patients without documented spirometry, chest computed tomography and other clinical information were used to define COPD. Tumor tissue DNA was extracted from formalin-fixed paraffin-embedded samples. DNA mutation analysis, multiplex immunohistochemistry (mIHC), calculation of tumor mutational burden (TMB), mutant-allele tumor heterogeneity (MATH), and predication of neoantigens were performed.

**Results:**

Although SNV mutations in lung cancer patients with COPD (G1 group) were generally higher than those in lung cancer patients without COPD (G2 group), the difference in the number of mutations was insignificant between the two groups. Of the 35 mutated genes, the number of them was higher in G1 than in G2, except that of EGFR. PI3K-Akt signaling pathway was enriched from significantly different genes. While TMB and MATH levels were not significantly different, the tumor neoantigen burdenwas markedly higher in G1 than that in G2. The level of CD68+ macrophages was significant higher in the stroma and total areas in the G1 group than in G2 group. The level of CD8+ lymphocytes was markedly higher in the stroma and showed a clear tendency forhigher expression in the G1 group than inthe G2 group. No significant differences were observed for the level of programmed death-ligand 1+ (PD-L1+), programmed death 1+ (PD-1+), and CD68PD-L1 in the stroma, tumor and total areas.

**Conclusion:**

Our study revealed different genetic aberrations and pathways, higher neoantigen burden, and higher level of CD68+ macrophages and CD8+ T lymphocytes in lung cancer patients with COPD. Our investigation implies that the existence of COPD should be considered and immunotherapy is a potential choice when treating lung cancer patients with COPD.

## Introduction

Chronic obstructive pulmonary disease (COPD) and lung cancer are leading cause of morbidity and mortality worldwide (1). The overall prevalence of spirometry-defined COPD is 8.6% among the general population aged 20 years or older in China (2). The incidence of these two diseases has been increasing and the trend is expected to continue. Previous studies have found that 40–70% of patients with lung cancer coexist with COPD, and the prevalence of lung cancer is significantly higher in patients with COPD than those without COPD (3, 4), and the presence of emphysema in COPD predicts a higher lung cancer risk adjusted for smoking status (5, 6). A recent large national cohort study from South Korea also pointed out that COPD is a strong independent risk factor for lung cancer in never smokers. Furthermore, never smokers with COPD had a similar risk of lung cancer compared with ever smokers without COPD (7).

The coexistence of COPD in lung cancer patients generally predicts a poor prognosis. In non-small cell lung cancer (NSCLC), the median survival time is shorter in patients with COPD than in those without COPD (8, 9). For surgical resection of lung cancer, the overall survival, and disease-free survival in the COPD group were significantly worse than those in the non-COPD group (10, 11). Patients with COPD undergoing lung cancer surgery were at higher risk of postoperative complications than patients with normal respiratory function (12).

Owing to the coexistence of COPD and lung cancer, traditional treatment options such as chemotherapy and tyrosine kinase inhibitors encounter more challenges in the clinic. The recent adoption of immunotherapy has shed lighton the NSCLC treatment. A few publications suggest that compared with non-COPD patients, lung cancer patients with COPD are more sensitive to immune checkpoint inhibitors (ICIs) and their prognosis can be improved by immunotherapies (13–15). Nevertheless, the investigation of mutations and the tumor immune microenvironment (TIME) in lung cancer patients with COPD remained understudied. Here, we reported the molecular altercations and TIME and their potential meanings in clinical settings.

## Materials and methods

### Patients

We performed a retrospective cohort study that included 435 pathologically confirmed lung cancer patients at the Ruijin Hospital from October 1^st^, 2018 to September 30^th^, 2021. Basic clinical information was collected, such as age, sex, smoking status, Eastern Cooperative Oncology Group (ECOG), tumor histology, pathological stage, number of metastatic sites and forced expiratory volume in 1 s (FEV1) predicted (FEV1%Pred). This study adhered strictly to the provisions of the Declaration of Helsinki (revised in 2013).

### Assessment of COPD

COPD was defined as a pre-bronchodilator FEV1/forced vital capacity (FVC) < 0.70. For those with documented spirometry, we used the Global Initiative for Chronic Obstructive Lung Disease (GOLD) criteria to assess the presence of COPD and to evaluate the severity of airflow obstruction (version 2018). The severity of airflow obstruction was staged by the GOLD criteria: mild (GOLD 1, FEV1 ≥80% predicted), moderate (GOLD 2, 50%≤ FEV1 <80% predicted), severe (GOLD 3, 30%≤ FEV1 <50% predicted), or very severe (GOLD 4, FEV1 < 30% predicted).

In our study, 435 patients were included; 362 patients had recorded spirometry while the remaining patients lacked such data. For patients with documented spirometry, the Global Initiative for Chronic Obstructive Lung Disease (GOLD) criteria were used to assess the presence of COPD. For patients without documented spirometry, we determined the presence of COPD based on the following criteria: emphysema or chronic bronchitis on chest computed tomography (CT) images, smoking history and clinical symptoms of cough, sputum, tightness and wheezing (16).

According to these criteria, the patients were divided into group 1 (G1: Lung cancer patients with COPD, N=141), and group 2 (G2: Lung cancer patients without COPD, N=294).

### DNA extraction

DTumor tissue DNA was extracted from formalin-fixed, paraffin-embedded samples using a TIANamp Genomic DNA Kit (TIANGEN, Beijing, China). Genomic DNA was extracted from the peripheral blood cells using the TGuide S32 Magnetic Blood Genomic DNA Kit (TIANGEN, China). The DNA concentration was measured using Qubit dsDNA HS Assay Kit (Themo Fisher Scientific, Waltham, MA, USA), and its quality was evaluated using Agilent 2100 BioAnalyzer (Agilent Technologies, Santa Clara, CA, USA).

### DNA mutation analysis

DNA libraries were constructed using 200 ng of DNA extracted from tumor tissues or peripheral blood cells using KAPA Hyper Preparation Kit (Kapa Biosystems, USA) according to the manufacturer’s instructions. Libraries were quantified using the AccuGreen High Sensitivity dsDNA Quantitation Kit (Biotium, USA), and the size of the libraries was determined by Agilent Bioanalyzer 2100 (Agilent, USA). Targeted regions were captured and hybridized using a 769-gene panel (Genecast Biotechnology Co., Ltd., Wuxi, China) and subsequently sequenced on the Illumina Nova seq platform after the libraries were purified and quantified. The clean reads were aligned to the human reference genome (Hg19, NCBI Build 37.5) using Burrows-Wheeler Aligner (v. 0.7.17) after removing low quality reads. Duplicated reads were removed using Picard toolkit (v. 2.1.0) (17) and realignment was performed by Genome Analysis ToolKit (v. 3.7) (18). Subsequently, theVarScan 2 program (19) was used for single nucleotide variation (SNV) calling, and ANNOVAR (20) was used for functional annotation of genetic variants. In order to identify somatic SNV and indel mutations, the somatic SNVs were selected if they did not fall into any one of the following filters: (i) located in intergenic regions or intronic regions; (ii) synonymous SNVs; (iii) depth < 40; (iv) allele frequency < 0.03; (vii) allele frequency ≥ 0.002 in the Exome Aggregation Consortum (ExAC) database. Subsequently, the identified tumor-related mutated genes were classified and subjected to Gene ontology (GO) analysis and Kyoto encyclopedia of genes and genomes (KEGG) pathway enrichment analyses.

### Calculation of tumor mutational burden and mutant-allele tumor heterogeneity

Tumor mutational burden (TMB) of the tumor tissue was calculated based on the absolute mutation counts of tumor samples against the mutation spots of the normal samples using the following formula: TMB=Absolute mutation counts*1000000/Total number of exonic bases (21). TMB was measured in mutations per megabase (Mut/Mb). With the Variant Allele Frequencies (VAF) determined by the ratio of alternate allele observations to the read depth at each site, we modified and calculated the mutant-allele tumor heterogeneity (MATH) score (22) to include all somatic variants with a VAF in a range from 0.02 to 1 using the following formula: MATH=100*median absolute deviation (MAD)/median of the VAF.

### Prediction of neoantigens

Based on the somatic SNVs and HLA typing of tumor tissues and paired control sample, neoantigens were predicted using netMHCpan-4.0 (23). To ensure accuracy, neoantigens with predicted binding affinities of mutation (Aff_mut) ≤ 500 and Aff_mut/Aff_wild< 1 were selected as the final results.

### Multiplex immunohistochemistry

An immune biomarker panel was used to quantitatively assess the levels of CD8+ cytotoxic T lymphocytes (CTLs), programmed death-1 (PD-1), programmed death ligand-1 (PD-L1), and CD68+ macrophages. Multiplex immunohistochemistry (mIHC) was performed using an Opal™ 7-color IHC Kit (PerkinElmer Inc., Boston, MA, USA) according to the manufacturer’s instructions. The antibodies used in this study included CD68 (ZM0060, dilution 1:500, Beijing Zhongshan Golden Bridge Biotechnology), and CD8 (ZA0508, dilution 1:100, Beijing Zhongshan Golden Bridge Biotechnology). The slides were incubated with the primary antibodies, followed by incubation in 0.3% hydrogen peroxide solution for blocking endogenous peroxidase. The fluorophores used were Opal 520, 540, 570, 620, 650, and 690. Nuclear counterstaining was conducted using DAPI. A Vectra 3.0.5 continuous spectrum imaging system (PerkinElmer Inc) and inForm 2.3.0 software (PerkinElmer Inc) were used to acquire and analyze images of tumor parenchyma (tumor), distant stroma and total regions.

### Statistical analysis

The Wilcoxon test was used to analyze TMB, MATH, neoantigen and immune cell infiltration between G1 and G2 group. Fisher’s exact test was used to analyze somatic mutations.

## Results

### Patient collection and baseline characteristics

There were 141 patients in G1 group and 294 patients in G2 group. Our analysis showed that age, sex, smoking status, tumor histology, pathological stage, number of metastatic sites FEV1/FVC, FEV1/FVC %pred, and FEV1%pred were different between G1 and G2 group. There was no difference in body mass index or family history of tumor between the G1 and G2 group. The clinical information of enrolled patients is listed in **Table 1**.

**Table 1:** Baseline clinical characteristics.

Characteristic	Total (N=435)	G 1: Lung cancer patients with COPD (N=141)	G 2: Lung cancer patients without COPD (N=294)	P
Age years, median	60.2±11.5	63.7±9.6	58.5±12.0	<0.0001
BMI	23.2±3.3	23.0±3.2	23.3±3.3	0.521
Gender				<0.0001
female	248(57.0%)	28(19.9%)	220(74.8%)	
Man	187(43.0%)	113(80.1%)	74(25.2%)	
Smoking status				<0.0001
Current Smoker	69(15.9%)	51(36.2%)	18(6.1%)	
Ever smoker	76(17.5%)	52(36.9%)	24(8.2%)	
Non-smoker	290(66.7%)	38(27.0%)	252(85.7%)	
Histology				<0.0001
Squamous carcinoma	25(5.7%)	17(12.1%)	8(2.7%)	
adenocarcinoma	399(91.7%)	115(81.6%)	284(96.6%)	
Small cell lung cancer	7(1.6%)	5(3.5%)	2(0.7%)	
others	4(0.9%)	4(2.8%)	0(0.0%)	
ECOG				1.000
0-1	434(99.8%)	141(100.0%)	293(99.7%)	
≥2	1(0.2%)	0(0.0%)	1(0.3%)	
Stage				0.001
I	272(62.5%)	70(49.6%)	202(68.7%)	
II	35(8.0%)	12(8.5%)	23(7.8%)	
II	58(13.3%)	26(18.4%)	32(10.9%)	
IV	70(16.1%)	33(23.4%)	37(12.6%)	
Number of metastatic sites, N(%)				0.016
No	367(84.4%)	109(77.3%)	258(87.8%)	
≤1	54(12.4%)	24(17.0%)	30(10.2%)	
>1	14(3.2%)	8(5.7%)	6(2.0%)	
Pulmonary function test (N=362)				
FEV1/FVC	78.8±10.1	69.7±10.0	83.4±6.2	<0.0001
FEV1/FVC %pred	99.6±11.8	89.3±12.5	104.8±7.1	<0.0001
FEV1 %pred	92.3±21.6	78.2±21.2	99.4±18.0	<0.0001
Family history of tumor				
Yes	14(3.2%)	8(5.7%)	6(2.0%)	0.086
No	421(96.8%)	133(94.3%)	288(98.0%)	

COPD, chronic obstructive pulmonary disease.

### Somatic mutations in lung cancer patients with COPD

**Figure 1:**
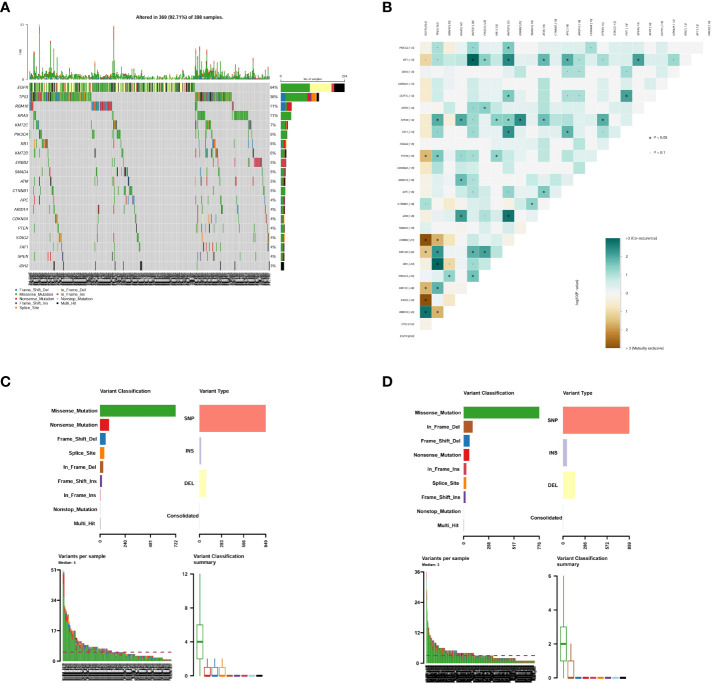
Summary of genetic aberrations in lung cancer patients. **(A)** Oncoplot shows the top 20 mutated genes in G1 and G2 group; **(B)** Co-occurring and exclusive mutated genes in G1 and G2 group; **(C)** SNV summary in G1 group; **(D)** SNV summary in G2 group. G1: lung cancer patients with COPD; G2: lung cancer patients without COPD; COPD, chronic obstructive pulmonary disease.

To investigate the molecular characteristics, we conducted targeted sequencing of tumors from the G1 and G2 group. Overall, we observed different SNV distribution between G1 and G2. Oncoplot analysis showed the top20 mutated genes with high frequencies in both groups (**Figure 1A**). A comprehensive heatmap of SNV mutations between G1 and G2 group is shown in **Supplementary Figure 1**. Both *EGFR* and *KRAS* are important driver mutations in lung cancer, however, they were mutually exclusive between G1 and G2 groups (**Figure 1B**). In G1, 957 mutations were detected in 141 patients with COPD, producing an average of 6.8 mutations (0-51 mutations) per patient. In G1, there were 722 missense mutations, 87 nonsense mutations, 85 deletions, and 21 insertions (**Figure 1C**). In G2, 1066 mutations were detected in 294 patients without COPD, producing an average of 3.6 mutations (0-36 mutations) per patient. In G2, there were 776 missense mutations, 58 nonsense mutations, 155 deletions, and 48 insertions (**Figure 1D**). However, no significant differences in the number of mutations were observed between the two groups.

There were total 46 gene fusions in the G1 and G2 groups, of which the G1 group harbored 8 gene fusions and the G2 group harbored 38. All gene fusions in G1 occurred only once. Gene fusion *EML4_13_ALK_20* was the most frequent fusion in G2, with a number of 7. Four gene fusions were shared between the G1 and G2 group: *RET_11_KIF5B_16*, *KIF5B_15_RET_12*, *EML4_6_ALK_20*, and *EML4_13_ALK_20*. Details of the gene fusion in the two groups are shown in **Table 2**.

**Table 2:** Distribution of gene fusions in G1 and G2 group.

Number of Gene fusions	1	2	3	4	5	6	7
G1 group	VTI1A_5_NTRK2_17 RET_11_NR1H4_8 RET_11_KIF5B_16 KIF5B_15_RET_12 FGFR3_17_TACC3_7 FGFR3_17_TACC3_6 EML4_6_ALK_20 EML4_13_ALK_20	None	None		None	None	
G2 group	SDC4_2_ROS1_32 ROS1_33_CD74_7 RET_11_KIF5B_16 RET_11_CCDC6_2 RET_10_C10orf11_4 KIF5B_16_RET_3 FLCN_4_MPRIP_4 EML4_7_RAB10_2 CD74_9_ROS1_32 CD74_8_ROS1_32 CD74_7_ROS1_32 CD74_6_ROS1_34 CD74_6_ROS1_33 CCDC6_1_RET_12 CCDC6_1_RET_11 ARFGAP3_9_EWSR1_10 ALK_19_EML4_7 ALK_19_EML4_6 ALK_18_EML4_6	KIF5B_15_RET_12 ETV6_4_NTRK3_15 EML4_20_ALK_20 AGBL4_6_NTRK2_16	None	EML4_6_ALK_20	None	None	EML4_13_ALK_20

G1: Lung cancer patients with COPD; G2: Lung cancer patients without COPD.

### Different mutation genes and pathway analysis

**Figure 2:**
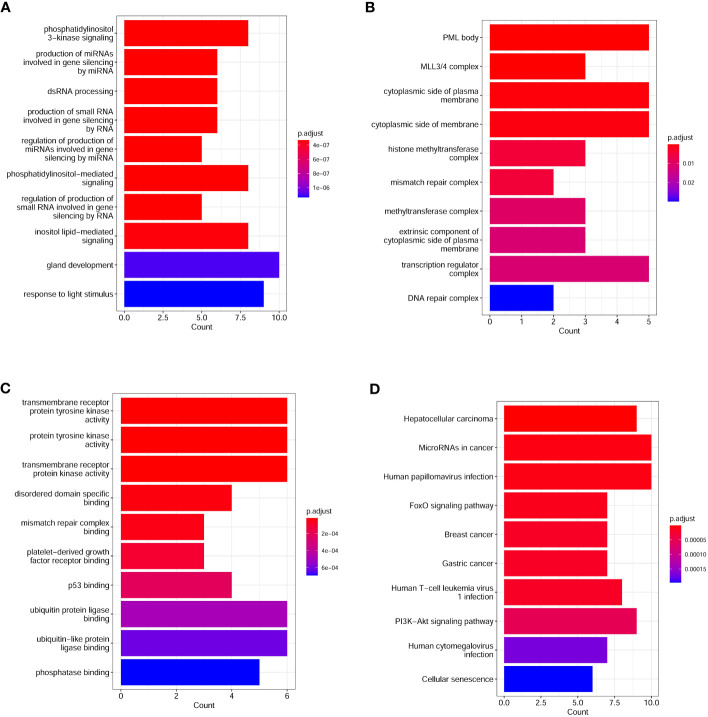
Top enriched GO and KEGG pathways in lung cancer patients. **(A)** Top10 biological processes enriched from differential genes between lung cancer patients with and without COPD; **(B)** Top10 cell components enriched from differential genes between lung cancer patients with and without COPD; **(C)** Top10 molecular functions enriched from differential genes between lung cancer patients with and without COPD; **(D)** Differential KEGG pathways between lung cancer patients with and without COPD. GO, gene ontology; KEGG, Kyoto encyclopedia of genes and genomes.

To investigate the number differences in mutated genes and pathways between the G1 and G2 group, we used Fisher’s exact test and found that there were 35 genes that were significantly different between the two groups (P < 0.05). Of which, the number of 35 mutated genes in G1 group was higher than those in G2 group. The mutated genes were *ERBB4*, *WT1*, *TP53*, *PTEN*, *AXIN1*, *STK11*, *SMAD2*, *KMT2D*, *PREX2*, *CREBBP*, *FAT1*, *KIT*, *LYN*, *PDGFRA*, *STAT3*, *FOXP1*, *MYCL*, *CARD11*, *NF1*, *GNAS*, *EGFR*, *MSH6*, *IRS2*, *RICTOR*, *ATR*, *NTRK3*, *KEAP1*, *KDM6A*, *KRAS*, *MYCN*, *MCL1*, *TERT*, *KMT2C*, *RB1*, and *PMS2*. The number of *EGFR*, however, was higher in the G2 group than in the G1 group. GO analysis showed that the top 10 biological processes enriched from significantly different genes between the G1 and G2 group included phosphatidylinositol 3-kinase signaling, production of miRNAs involved in gene silencing by miRNA, phosphatidylinositol-mediated signaling, regulation of production of small RNA involved in gene silencing by RNA, inositol lipid-mediated signaling, and response to light stimulus (**Figure 2A**). Top 10 cell components enriched from significantly different genes between G1 and G2 group included histone methyltransferase complex, mismatch repair complex, transcription regulator complex, and DNA repair complex (**Figure 2B**). Top 10 molecular functions enriched from significantly different genes between G1 and G2 group included transmembrane receptor protein tyrosine kinase activity, protein tyrosine kinase activity, transmembrane receptor protein kinase activity, platelet-derived growth factor receptor binding, p53 binding, and ubiquitin protein ligase binding (**Figure 2C**). KEGG analysis between G1 and G2 group showed that the top 10 pathways enriched from significantly different genes include Human papillomavirus infection, FoxO signaling pathway, Human T-cell leukemia virus 1 infection, and PI3K-Akt signaling (**Figure 2D**).

**Figure 3:**
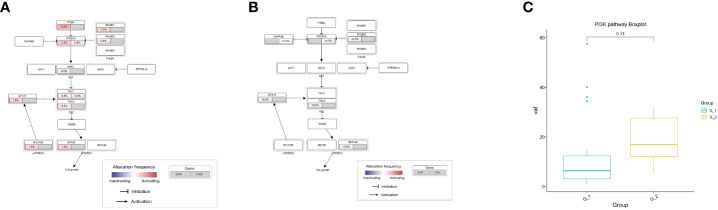
Analysis of PI3K-Akt signaling pathway in lung cancer patients. **(A)** Mutation frequencies of SNV and CNV enrich in PI3K-Akt signaling pathway in G1 group; **(B)** Mutation frequencies of SNV and CNV enrich in PI3K-Akt signaling pathway in G2 group; **(C)** Comparison of variant allele frequency in PI3K-Akt signaling pathway between G1 and G2 group; G1: lung cancer patients with COPD; G2: lung cancer patients without COPD. COPD, chronic obstructive pulmonary disease.

We compared the mutated genes in the PI3K-Akt signaling pathway. We only considered missense mutations, loss-of-function mutations, and copy number variation. We found that the mutational load of SNVs in the G1 group was higher than in the G2 group (**Figures 3A, B**). However, the median variant allele frequency (VAF) of SNVs in the PI3K-Akt signaling pathway genes was not significant in the G1 group (**Figure 3C**).

### TMB, MATH and neoantigen analysis

**Figure 4:**
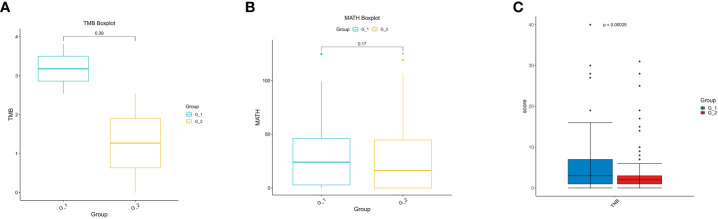
Analysis of TMB, MATH and TNB in lung cancer patients. **(A)** Analysis of tumor mutation burden (TMB); **(B)** Analysis of mutant-allele tumor heterogeneity analysis (MATH); **(C)** Analysis of tumor neo-antigen burden (TNB).

TMB is regarded as a predictive biomarker of the response to immune therapy. MATH is used to quantify differences in the dispersion or spread of allele frequencies; its quantitative nature allows the assessment of genetic heterogeneity and acts as an actionable biomarker for cancer treatment. Mutant allele frequency distributions in cancer samples were also used to estimate intratumoral heterogeneity and its implications for patient survival. We then delineated the differences in TMB and MATH between the G1 and G2 groups and found no differences in TMB (**Figure 4A**) or MATH (**Figure 4B**).

Cancer neoantigens are produced from newly emerging gene mutations in cancer cells and are potential targets for T cell-mediated anti-tumor cytotoxicity. Patients who produce more neoantigens are more likely to benefit from immunotherapies. We analyzed the neoantigens and found that they were significantly higher in G1 than in G2 (p = 0.00025) (**Figure 4C**). Our results indicate that immunotherapy can be used in lung cancer patients coupled with COPD.

### Investigation of tumor immune microenvironment

**Figure 5:**
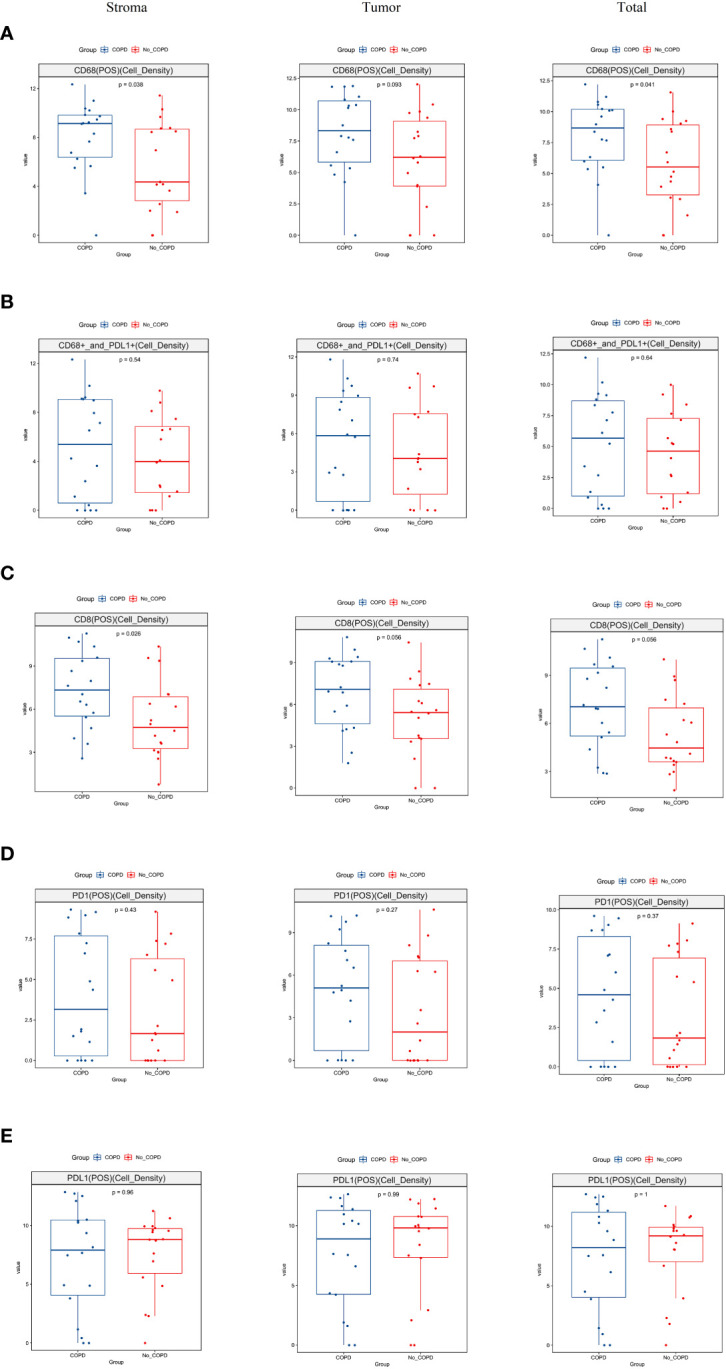
Analysis of tumor immune microenvironment in lung cancer patients by multiplex immunohistochemistry. **(A)** The distribution of CD68 macrophages was significantly higher in stroma (p = 0.038) and total regions (p = 0.041) and showed a trend to significance in tumor region (p = 0.093) in lung cancer patients with COPD than those without COPD; **(B)** The distribution of CD68 and PD-L1 co-expressed lymphocytes was no significant difference in the stroma (p = 0.54), tumor (p = 0.74) and total (p = 0.64) regions between lung cancer patients with COPD and without COPD; **(C)** The distribution of CD8+ lymphocytes was significantly higher in stroma region in lung cancer patients with COPD than those without COPD (p = 0.026); the distributions in the tumor region (p = 0.056) and total region (p = 0.056) were barely statistically insignificant in lung cancer patients with COPD than those without COPD patients; **(D)** The distribution of PD-1+ lymphocytes was insignificant in the stroma, tumor and total regions between lung cancer patients with COPD and those without COPD; **(E)**. The distribution of PD-L1+ lymphocytes was insignificant in the stroma, tumor and total regions between lung cancer patients with COPD and those without COPD. COPD, chronic obstructive pulmonary disease.

To deconvolute the TIME in lung cancer patients with COPD, we investigated PD-1, PD-L1, CD8 T lymphocytes, and CD68 macrophage in the stroma and tumor areas. CD68+ macrophages in the stroma and total areas were markedly higher in COPD patients than in non-COPD patients; although the difference was not significant in the tumor area, it showed a favorable statistical trend in COPD patients compared to non-COPD patients (**Figure 5A**). There was no significant difference in CD68+PD-L1+ lymphocytes in the stroma, tumor and total areas between COPD and non-COPD patients (**Figure 5B**). The cell density of CD8+ lymphocytes in the stroma area, was significantly higherin COPD than in non-COPD patients; the differences in the tumor and total areas were close to significance in COPD patients than in non-COPD patients (**Figure 5C**). For PD-1+ and PD-L1+ lymphocytes, there were no significant differences in the stroma, tumor, or total areas between COPD and non-COPD patients (**Figures 5D, E**).

## Discussion

COPD is a common comorbid disease in lung cancer. It is estimated to affect 40-70% of lung cancer patients, depending on diagnostic criteria (3, 4). However, few investigations have been conducted on the molecular characteristics in lung cancer patients with COPD. In this study, we reported the genetic mutations and TIME in patients with lung cancer and concomitant COPD. We found significantly different SNVs and pathways, neoantigens, and TIME between lung cancer patients with and without COPD, implying different treatment strategy in clinical practice.

Profiling of tumor tissues revealed that molecular alterations occurred frequently in lung cancer patients with COPD. In our study, we identified the number of 35 mutated genes was significantly higher in G1 than in G2 group. Of these, we found that the number of the well-recognized driver mutation genes *KRAS*, and *NTRK3* were significantly higher in lung cancer patients with COPD than in those without COPD, while the number of *EGFR* showed an opposite trend, in accordance with the results of He et al. (24). This may be attributed to the higher proportion of women, non-smokers and adenocarcinoma in the G2 group. Sex, adenocarcinoma histology, and never-smoking status are considered the most important factors associated with *EGFR* mutation (25, 26). We found that the number of tumor suppressor genes *RB1*, *TP53*, and *PTEN* was also significantly higher in patients with COPD than in patients without COPD. Multiple studies have found that mutations in *RB1* and *TP53* are closely related to the transformation from lung adenocarcinoma to small cell lung cancer, which often occurs in patients receiving TKIs therapy and is associated with a relatively poor prognosis (27, 28), suggesting that TKIs therapy might not be a good choice for lung cancer patients with COPD. *PIK3CA* mutations were more in G1 than in G2, which is in accordance with the investigation by Hirata et al. (29). In our study, the PI3K-Akt signaling pathway was enriched from significantly different genes. Activating mutations of*PIK3CA* were associated with increased PI3K activity in lung cancer (30, 31). *PIK3CA* mutation plays a role on lung tumorigenesis as a powerful promoter that is initiated by other oncoproteins, particularly *KRAS* or *BRAF* mutation, and not as an initiator in itself (32). Our findings suggest that the PI3K-Akt signaling pathway stimulates the development of lung cancer and that COPD is at a pre-cancer stage (33). Overall, the presence of COPD affects the types and proportions of tumor gene mutations, and these differences may be related to the poor survival prognosis of lung cancer patients with COPD. However, the causal relationship requires further investigation.

Neoantigens are linked to DNA repair mutations and generate increased numbers of tumor-infiltrating lymphocytes (TILs). Neoantigens correlate with increased expression of multiple proinflammatory cytokines and immune-related genes, M1-polarized macrophage genes, programmed cell death ligand-1 (PD-L1) and programmed cell death-1 (PD-1) (34, 35). We observed that lung cancer patients with COPD had a significantly higher neoantigen loads than those without COPD (p = 0.00025), indicating that they likely benefit from immune-related therapies. This finding is meaningful in clinical setting and warrants further investigation.

The recent development of ICIs has revolutionized lung cancer treatments. Compared to lung cancer patients without COPD, lung cancer patients with COPD have better benefits when treated with ICIs. Lee et al. investigated the clinical impact of COPD on the treatment response to pembrolizumab in patients with non-small-cell lung cancer and concluded that patients with COPD had a higher response rate and improved overall survival (OS) and progress-free survival (PFS) (36). Biton et al. observed a longer PFS in 39 advanced NSCLC patients with COPD treated with nivolumab (37). Zhou et al. suggested that the presence of COPD in advanced lung cancer patients was associated with better survival and longer PFS (16). To characterize the TIME and elucidate its potential mechanism in lung cancer patients with COPD, we assessed the TIME using mIHC. Our investigation showed that the cell density of CD68+ macrophages was significantly higher and that CD8+ T lymphocytes showed a clear tendency to be more significant in lung cancer patients with COPD than in those without COPD. Mark et al. used flow cytometry and T-cell receptor sequencing to profile immune cell subtypes and investigated the effects of COPD on non-small-cell lung cancer (14). They observed increased numbers of CD8+ and CD4+ lymphocytes in the lungs of patients with COPD. Biton et al. also found that COPD is associated with an increased sensitivity of CD8+ tumor-infiltrating T lymphocytes in tumors, suggesting a higher sensitivity to PD-1 blockade in patients with COPD (37). These findings are consistent with those of previous studies. Lee et al. proposed that increased tumor infiltrating lymphocytes in patients could be related to higher tumor mutation burden. However, the results of our study did not support this hypothesis.

Increased inflammatory cells (CD8+ cells and macrophages) were reported in airways in patients with COPD (38). Tumor-associated macrophages (TAM) are generally regarded as poor prognostic marker in human cancers (39). However, in the tumor microenvironment of NSCLC, the high density of PD-L1+TAM in tumor tissue predicted better survival in NSCLC patients who were treated with PD-1/PD-L1 inhibitors (40, 41). In our case, the density of CD68+PD-L1+ macrophages was higher in the G1 than in the G2 group, suggesting potential benefits from immunotherapy, though the difference was not significant. Our study suggests that the density of CD68+ macrophage is significantly higher in patients with COPD. Macrophages are very polarized and their phenotypes may change between pro-inflammatory M1 and anti-inflammatory M2. In this study, the phenotypes of M1 and M2 were not investigated and it warrants investigations in the future. As COPD is a chronic airway inflammatory disease characterized by the infiltration of CD8+ T lymphocytes and CD68+ macrophages (42), we concluded that the differences in CD8+ T lymphocytes and CD68+ macrophages between the two groups may have been caused by COPD itself, suggesting that the existence of COPD is an important factor for the benefit of immunotherapy.

This was a retrospective clinical study conducted in the real world, and the nature of the study restricts its power. This study has limitations. First, the two groups differ in terms of age, sex, and smoking history, which may lead to differences in gene mutations and the tumor microenvironment. Here, we analyzed differences from the perspective of COPD. Although inadequate, this study revealed the impact of COPD on lung cancer, suggesting that COPD should be considered when treating lung cancer. Second, the association between COPD in lung cancer patients and the potential benefits of PFS or OS under immunotherapy treatment has not been validated in clinical settings. Most of the patients treated in our study received chemotherapy or targeted therapy. Only a few patients have undergone immunotherapy. Therefore, a prospective study of the association between COPD and immunotherapy in lung cancer patients should be conducted.

In conclusion, we investigated the molecular landscape and TIME in lung cancer patients with COPD. Our study suggests different genetic alterations, pathways, higher neoantigen burden and the presence of higher density of CD68+ macrophages and CD8+ T lymphocytes in lung cancer patients with COPD. Our investigation implies that the presence of COPD is an important factor for the benefit of immunotherapy in lung cancer patients.

## Data availability statement

The datasets presented in this study can be found in online repositories. The names of the repository/repositories and accession number(s) can be found in the article/**Supplementary Material**.

## Ethics statement

The studies involving human participants were reviewed and approved by Ruijin Hospital Ethics Committee Confidential Agreement. The patients/participants provided their written informed consent to participate in this study.

## Author contributions

All authors made a significant contribution to the work reported, whether that is in the conception, study design, execution, acquisition of data, analysis and interpretation, or in all these areas; took part in drafting, revising or critically reviewing the article; gave final approval of the version to be published; have agreed on the journal to which the article has been submitted; and agree to be accountable for all aspects of the work. All authors contributed to the article and approved the submitted version.
